# Dual-task training with progression from variable- to fixed-priority instructions versus dual-task training with variable-priority on gait speed in community-dwelling older adults: A protocol for a randomized controlled trial: Variable- and fixed-priority dual-task for older adults

**DOI:** 10.1186/s12877-020-1479-2

**Published:** 2020-02-22

**Authors:** Francis Trombini-Souza, Marcelo de Maio Nascimento, Tarcísio Fulgêncio Alves da Silva, Rodrigo Cappato de Araújo, Mônica Rodrigues Perracini, Isabel C. N. Sacco

**Affiliations:** 1 Department of Physical Therapy, University of Pernambuco (UPE) Campus Petrolina, Petrolina, PE Brazil; 2 Department of Physical Education, Federal University of Sao Francisco Valley (UNIVASF) Campus Petrolina, Petrolina, PE Brazil; 3 Master’s and Doctoral Programs in Physical Therapy, Universidade Cidade de São Paulo, São Paulo, SP Brazil; 4 Physical Therapy, Speech and Occupational Therapy Department, Universidade de Sao Paulo (USP), School of Medicine, São Paulo, SP Brazil

**Keywords:** Falls, Older adults, Dual task, Balance training, Functional activities

## Abstract

**Background:**

Functional independence and safe mobility, especially in older people, mostly rely on the ability to perform dual tasks, particularly during activities with variable- and fixed-priority attention. The aim of this study is to compare the dual-task training with progression from variable- to fixed-priority instructions versus dual-task training with variable-priority on gait speed in community-dwelling older adults.

**Methods:**

This is an assessor- and participant-blinded, two-arm, randomized controlled trial with 60 community-dwelling male and female older adults between the ages of 60 and 80 years old. Participants will be randomly allocated into either the intervention group or the control group using a computer-generated permuted block randomization schedule. The intervention group will undertake a progressive dual-task training in which the participants will be progressively submitted to dual-task walking and postural balance exercises with variable- to fixed-priority instructions. The control group will be submitted to dual-task training with variable-priority attention exercises. Both groups will receive 48 sessions lasting for 60 min each over 24 weeks. The primary outcome will be the gait speed under single- and dual-task conditions. Secondary outcomes will include spatiotemporal gait parameters, functional balance, executive function, falls, quality of life, and depression symptoms. All the analyses will be based on the intention-to-treat principle.

**Discussion:**

This is the first assessor- and participant-blinded, two-arm, randomized controlled trial with 6 months of intervention and an additional 6-month post-training follow up aiming to evaluate the effectiveness of training with progression from variable- to fixed-priority instructions on gait biomechanics, postural balance, falls episodes, executive functioning, and quality of life in community-dwelling older adults. If our hypotheses are confirmed, this training protocol can be implemented widely to improve gait speed and other functional activities and quality of life in community-dwelling older adults. This study protocol can be used to improve these functional aspects of community-dwelling older adults. This study may also contribute to future guidelines for the improvement of these clinical and biomechanical aspects in older people.

**Trial registration:**

ClinicalTrials.gov Identifier - NCT03886805, Registered 22 March 2019.

## Background

Older adults have an increased risk of falling, especially when they are required to perform a concurrent cognitive or secondary motor task while walking and carrying objects or paying attention to traffic [1–3]. In community-dwelling older adults this type of dual-task activity also significantly reduces gait speed [4] and increases gait variability [5–9]. Dual-task activities can be performed by shifting attention between tasks (dual task with variable-priority instructions) or placing equal amounts of attention on both tasks (dual task fixed-priority instructions) [10].

Regarding dual-task instruction priority, the literature has shown a bit more effect of training under variable- than fixed-priority instructions, although both are considered to have a great effect size [11]. These results are likely why participants trained under variable-priority instruction are able to learn faster and retain instruction better than dual-task training with fixed-priority instruction [12]. Nevertheless, we must consider that motor and cognitive tasks are often and simultaneously demanded in everyday situations, and so these tasks should be trained in protocols aiming for a dynamic postural balance in older adults. In addition, we can assume that fixed-priority instructions could be adopted as a progression of the variable-priority method since the learning and retention of the simultaneous motor and cognitive tasks of the former method appear more complex than the latter. Based on this rationale, in the first 12 weeks of training, both groups will be trained with dual-task activities exclusively under variable-priority instructions so that they can better learn and retain the motor and cognitive gains provided by this type of dual-task training, as already shown in the literature [12]. Over the next 12 weeks, only the participants in the control group will continue to evolve into the dual-priority variable task training. The experimental group will receive an exclusively dual-task training with fixed-priority, to better mimic most of the functional activities of daily living. To our knowledge, no research to date has attempted to prove this rationale.

In addition, although short-term benefits of dual-task exercises are known, the required frequency, duration, and intensity of training programs and need for supervision are still inconclusive [13], especially on static and dynamic postural stability [11]. Furthermore, there is low methodological quality among the existing studies regarding short-term follow ups [14].

Therefore, this protocol for a 6-month controlled trial with a 6-month follow up post-training will examine whether a dual-task protocol training with progression from variable- to fixed-priority instructions is more effective than only variable-priority dual-task protocol training for improving gait speed in community-dwelling older adults. We hypothesize that the experimental group’s (EG) participants who receive the proposed protocol (dual tasks with variable- and fixed-priority instructions) will achieve better improvements regarding the studied outcomes in comparison to the control group (CG) undertaking just dual tasks with variable-priority training.

## Methods/design

### Study design

This is an assessor- and participant-blinded, two-arm, randomized controlled superiority trial. The study has been written according to the recommendations of the World Health Organization, the International Committee of Medical Journal Editors, the Consolidated Standards of Reporting Trials (CONSORT) [15], and the Standard Protocol Items (SPIRIT) statements [16, 17]. This trial was prospectively registered at ClinicalTrials.gov (NCT03886805).

**Fig. 1:**
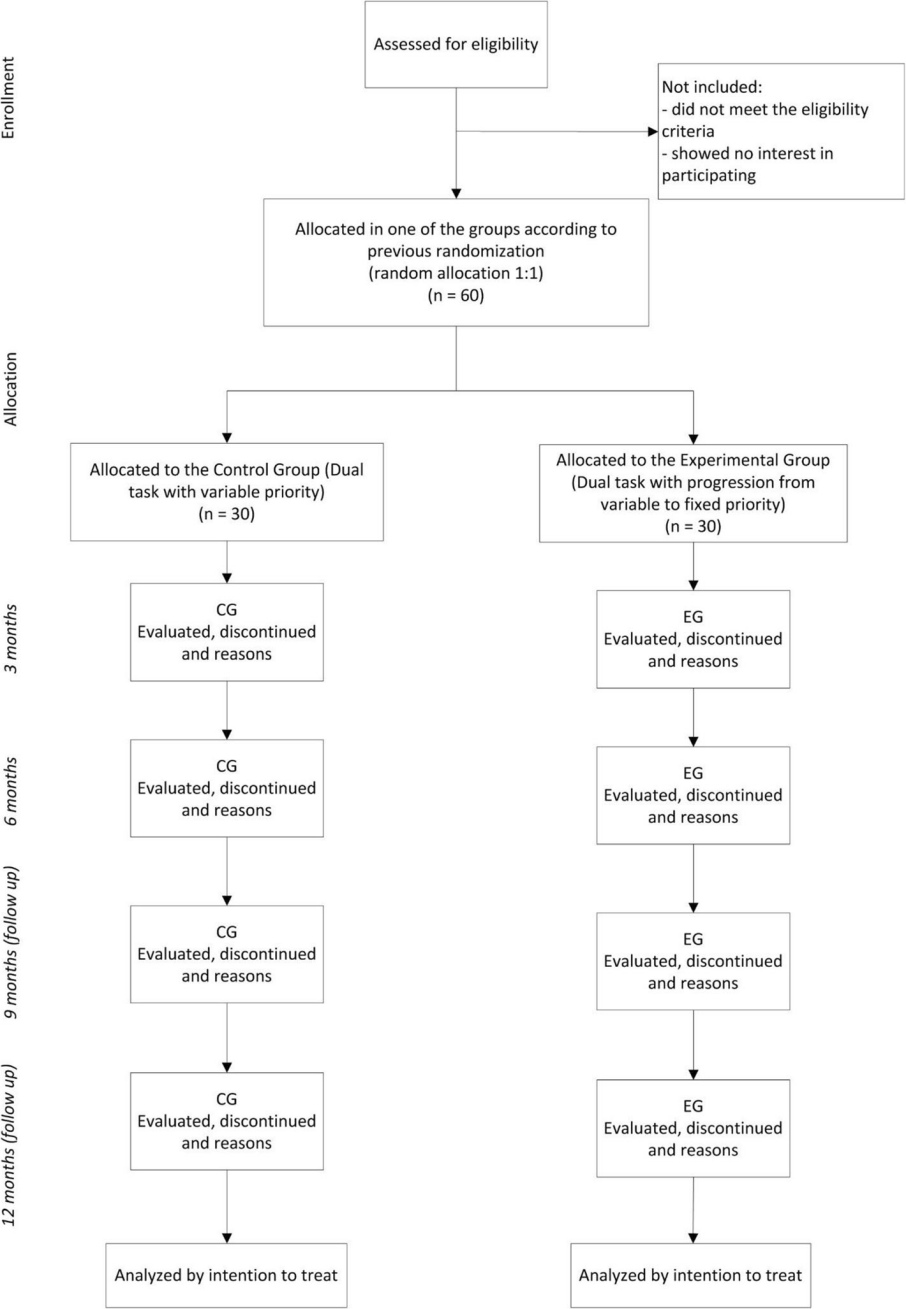
Flowchart of the study

Sixty community-dwelling older adults between the ages of 60 and 80 years old will be randomly assigned to either dual-task training with activities progression from variable- to fixed-priority attention (EG) or to dual-task training with variable-priority attention (CG) for 6 months. The assessments will be performed at baseline (T1), at 3 months of intervention (T2), at the end of the 6 months of intervention (T3), as well as at 3 (T4) and 6 (T5) months post-intervention (Fig. 1). The T2 assessment was established in order to verify any change after the period when the EG will be trained only with fixed-priority instructions and the CG will be keeping trained with variable priority instructions.

### Study settings

The study data collection will be carried out in the Laboratory of Human Biomechanics and Functional Activity of the Department of Physical Therapy at the University of Pernambuco *Campus* Petrolina, Brazil. The protocol training will be carried out in the multi-sport gym of the Department of Physical Education, at the Federal University of Sao Francisco Valley, Brazil.

### Ethics approval, consent to participate and dissemination

This trial was approved by the Ethics Committee of the University of Pernambuco (CAAE: 71192017.0.0000.5207). All patients will be asked to provide written informed consent prior to randomization, using standard forms.

### Sample size

Gait speed (primary outcome) under fixed-priority dual tasks was used to calculate the sample size [18]. We adopted a minimal clinically important difference of 0.05 m/s, an effect size of 0.20 [19], a power of 95% (1 - β), an alpha of 0.05, and a design of *F*-statistic repeated measures with a within- and between-subjects interaction effect. Forty-eight participants were initially obtained as the study sample total. Taking into account a 20% sample loss, 60 participants will be assessed and allocated to the study by a ratio of 1:1. The sample size was calculated using the G*Power 3 [20].

### Participants and recruitment

Participants will be recruited from community health centers and other settings that have specialized health care for older people, such as parks, squares, and churches in the city. Announcements on radios, local newspapers, and social networks (Facebook and Instagram) will be used. All participants will sign a consent form before starting the assessments.

### Eligibility criteria

Participants of both sexes between the ages of 60 and 80 years will be included in this study. For security reasons, only individuals presenting a score of ≥52 (up to a maximum of 56) on the Berg Balance Scale [18], a score of ≥24 (up to a maximum of 30) on the Mini-Mental State Examination [21], and who are able to walk uninterrupted for a distance of 10 m at a self-selected velocity of at least 1 m/s without assistance from another person, cane, or walker will be included. Potential participants will be excluded if they (i) have any contraindication to postural balance and cognitive exercise, (ii) have fallen two or more times in the last 12 months, (iii) participate or have participated in any regular or structured physical exercise program two or more times per week in the last 6 months, (iv) have a chronic health condition for which exercise is contraindicated, (v) have had any upper or lower limb fracture in the last 6 months [22], (vi) have evidence of any surgical procedures to the knees, ankles, or hips or have had muscle damage in the last 6 months [23], (vii) have self-reported uncontrolled diabetes, (viii) no able to speak and understand the Portuguese language, and (ix) refusal to give informed consent.

### Concealed allocation

The concealed randomization system schedule will be prepared by an independent researcher (TFAS) who is not aware of the numeric codes for the EG and CG. The numerical sequence will be maintained in opaque envelopes sequentially numbered from 1 to 60, following the order generated by the software. The randomization procedure will follow the instructions described in the literature [24]. The code sequence will be kept confidential and stored in a location that blind assessors, participants, and the statistician of this clinical trial will not have access to until the end of the study.

### Blinding

Participants, assessors and the statistician will be blinded regarding the group allocation. All assessments of the study outcomes will be conducted by assessors blinded to group allocation. Considering that both groups will undergo variable-priority activities at some stages of the training protocol, participants will not have the ability to differentiate which group they are allocated to. Thus, we consider participants to be blind to allocation. On the other hand, due to the nature of the intervention, physical therapists cannot be blinded to allocation. However, they will be strongly encouraged to not disclose the allocation status of the participants at the follow-up training [25].

Before each assessment, all participants will be instructed to not disclose what type of training they are receiving. Code breaks should occur only in exceptional circumstances when knowledge of the dual-task balance protocol training is essential for further management of the participant.

### Intervention

The EG will be trained with variable-priority instruction activities during the first 3 months (T1 to T2) and in the subsequent 3 months (T2 to T3) will be submitted to training with fixed-priority instructions activities. The GC will be trained under variable-priority instruction activities over the 6 months (T1 to T3). The detailed evolution of each of the groups can be found in Tables 1 and 2.

**Table 1:** Detailed conceptual basis regarding foci, tasks, and respective progressions to structure the training protocol

Focus	Strategy	Task
1. Postural balance	a) Change the base of support	**Standing** 1) Normal standing (bipodal support with feet separated at shoulder width 2) Feet together (bipodal support with feet side by side) 3) Semi-tandem stance 4) Tandem stance 5) One leg support on the dominant leg 6) One leg support on the non-dominant leg
		**Walking** 7) Semi-tandem 8) Tandem
	b) Displace the center of mass in different directions and with different bases of support	1) Sequentially perform F1a (1–6) by first laterally moving the trunk with shoulder abducted at least above 60 degrees 2) Sequentially perform F1a (1–6) by first laterally moving the trunk with the arms alongside the body
	c) Reach the body limits of stability and holding them at this point	1) Sequentially perform F1a (1–6) by first laterally moving the trunk with shoulder abducted at least above 60 degrees 2) Sequentially perform F1a (1–6) by first laterally moving the trunk with the arms alongside the body
	d) Change the somatosensation input (stability/complacency of the support surface)	Standing on different surfaces (rigid, soft or rough) 1) Sequentially perform F1a (1–6)
		Walking on different surfaces (rigid, soft or rough) 2) Sequentially perform F1a (7–8)
	e) Change the vision input	Standing with eyes closed 1) Sequentially perform F1a (1–6)
		Walking with eyes closed 2) Sequentially perform F1a (7–8)
2. Muscle strength and power, and postural balance during stationary activities performance	a) Standing still in a semi-squatting posture	1) Increase the time staying in this posture
	b) Weight-loaded in a single-leg stance	1) Gradually increase the standing time in this posture
	c) Sit to stand	1) Increase the number of cycles per seconds (starting at a frequency of one cycle per second)
3. Pattern, agility, power, response time, and postural balance during walking	a) Increase the stride length and walking speed	1) Gradually increase the stride length using hula hoops, traffic cones, climbing ladder, marking and boxes positioned on the floor 2) Gradually increase the walking speed 3) Gradually increase and decrease the forward and backward walking acceleration while a command (rapid or slow) is proffered by the instructor
	b) Change the walking directions	1) Forward, backward, left, right, and turning around 2) Gradually reduce the interchange time between different directions 3) Spinning and spinning-walking
	c) Change the stability/complacency of the support surface	1) Sequentially perform F1a (7–8) first on a stable surface (floor) and then on an unstable one (foam cushion or mats) 2) Randomly perform F1a (7–8) first on a stable surface (floor) and then on an unstable one (foam cushion or mats)
	d) Change the level of walking	1) Increase step, obstacle, and ramp negotiation and the alternance between the obstacles
4. Secondary motor tasks performance	a) Manipulative tasks while walking	1) Closing buttons and zippers while walking; taking the wallet, handkerchief, or coins out pocket 2) Transportation and manipulation of the object
	b) Coordination tasks while walking	1) Contour a ball around the own trunk 2) Bouncing a ball on the ground 3) Clap the hands according to a command
	c) Confidence in balance-required tasks	1) While in a stationary posture, achieve and pick up objects from a raised height 2) While walking, bend laterally and/or anteriorly the trunk at the limits of stability in order to achieve and pick up objects with different sizes, quantities, and locations (on a bench or on the ground)
	d) Maneuvering traffic	1) Avoiding collision with the other participants of the group while walking
5. Cognitive tasks	a) Working memory	1) Phonological fluency: Say words according to the letter they begin with (such as” F″,” A”, or” S″) 2) Semantic fluency: Say words according to a category (such as” animals”,” fruits” or “supermarket shopping list”, and sometimes” verbs” v” nouns”), randomly chosen by the instructor 3) N-back and n-forward problem solving (e.g., subtracting or summing *n* from an established number) 4) Auditory forward digit span: Remember as many as possible of the number/letter forward sequence, you were told 5) Auditory backward digit span: Remember as many as possible of the number/letter back sequence, you were told 6) Visual forward digit span: Remember as many as possible of the number forward sequence shown to you by means of cardboard 7) Visual backward digit span: Remember as many as possible of the number/letters backward sequence shown to you by means of cardboard
	b) Perceptual performance	1) Quickly count the amount of objects/dots shown in a paperboard 2) Quickly identify the number of points/objects on a paperboard (among other different objects shown on the data show projection on the wall)
	c) Mental tracking	1) Count (and say after) how many times a word (e.g. “cat”) was said in a story about this subject (cats)
	d) Reaction time	1) React as fast as possible (e.g. clap the hands, or say “yes”) when a certain word was said or sound was emitted 2) React as fast as possible (e.g. clap the hands, or say “yes”) when a cardboard with a dot, figure or letter was shown 3) Go/No-go visual reaction time (walk when a “go” cardboard was shown and stop walking when a “stop” cardboard was shown)
	e) Selective reaction time	1) Say “yes” when hearing “strawberry” but say nothing when hearing another sort of fruit 2) Increase walking speed when hearing “orange”, decrease walking speed when hearing “banana”, say “yes” when hearing “strawberry”, but say nothing or change the walking condition when hearing another sort of fruit
	f) Executive performance	1) Perform steps according to arrows directions: identify the direction of the arrow tips and perform step as fast as she/he can 2) Color reading interference (a variant of the classic Stroop test): Say the printed color and not the color name that the word was printed (and presented in the wall by data show projection) 3) View the arrow and commands direction projected on a screen by means a data show equipment and quickly transport yourself to the presented direction /command 4) Simultaneously pronouncing and stepping on numbers stamped on a rug

**Table 2:** Detailed scheme on the association between the foci, strategies, and task training for the control and experimental groups

Stage	Timeline (week)	Foci, strategies, and tasks	
		CG	EG
1	1st to 3rd	**F1**a (1–8), **F1**b (1–2), **F1**c (1–2), **F2**a (1), **F2**b (1), **F2**c (1), **F3**a (1–2), **F3**b (1), **F3**c (1), **F5**a (1–2)^**a**^	Idem to the CG
2	4th to 6th	**F1**a (1–8), **F1**b (1–2), **F1**c (1–2), **F1**d (1–2), **F1**e (1), **F2**a (1), **F2**b (1), **F2**c (1), **F3**a (1–3), **F3**b (1–2), **F3**c (1–2), **F4**a (1–2)^**a**^, **F4**b (1)^**a**^, **F5**a (1–3)^**a**^	Idem to the CG
3	7th to 9th	**F1**a (1–8), **F1**b (1–2), **F1**c (1–2), **F1**d (1–2), **F1**e (1–2), **F2**a (1), **F2**b (1), **F2**c (1), **F3**a (1–3), **F3**b (1–2), **F3**c (1–2), **F3**d (1), **F4**a (1–2)^**a**^, **F4**b (1–2)^**a**^, **F5**a (1–3), **F5**d (1–3)^**a**^	Idem to the CG
4	10th to 12th	**F1**a (1–8), **F1**b (1–2), **F1**c (1–2), **F1**d (1–2), **F1**e (1–2), **F2**a (1), **F2**b (1), **F2**c (1), **F3**a (1–3), **F3**b (1–3), **F3**c (1–2), **F3**d (1), **F4**a (1–2)^**a**^, **F4**b (1–3)^**a**^, **F4**c (1)^**a**^, **F5**a (1–4)^**a**^, **F5**e (1–2)^**a**^	Idem to the CG
5	13th to 15th	**F1**a (1–8), **F1**b (1–2), **F1**c (1–2), **F1**d (1–2), **F1**e (1–2), **F2**a (1), **F2**b (1), **F2**c (1), **F3**a (1–3), **F3**b (1–3), **F3**c (1–2), **F3**d (1), **F4**a (1–2)^**a**^, **F4**b (1–3)^**a**^, **F4**c (1–2)^**a**^, **F5**a (1–7)^**a**^, **F5**b (1)^**a**^, **F5**e (1–2)^a^	**F1**a (1–8), **F1**b (1–2), **F1**c (1–2), **F1**d (1–2), **F1**e (1–2), **F2**a (1), **F2**b (1), **F2**c (1), **F3**a (1–3), **F3**b (1–3), **F3**c (1–2), **F3**d (1), **F4**a (1–2)^**b**^, **F4**b (1–3)^**b**^ **F4**c (1–2)^**b**^, **F5**a (1–7)^**b**^, **F5**b (1)^**b**^, **F5**e (1–2)^**b**^
6	16th to 18th	**F1**a (1–8), **F1**b (1–2), **F1**c (1–2), **F1**d (1–2), **F1**e (1–2), **F2**a (1), **F2**b (1), **F2**c (1), **F3**a (1–3), **F3**b (1–3), **F3**c (1–2), **F3**d (1), **F4**a (1–2)^**a**^, **F4**b (1–3)^**a**^, **F4**c (1–2)^**a**^, **F5**a (1–7)^**a**^, **F5**b (1–2)^**a**^, **F5**c (1)^**a**^, **F5**d (1–2)^**a**^, **F5**e (1–2)*	**F1**a (1–8), **F1**b (1–2), **F1**c (1–2), **F1**d (1–2), **F1**e (1–2), **F2**a (1), **F2**b (1), **F2**c (1), **F3**a (1–3), **F3**b (1–3), **F3**c (1–2), **F3**d (1), **F4**a (1–2)^**b**^, **F4**b (1–3)^**b**^, **F4**c (1–2)^**b**^, **F5**a (1–7)^**§**^, **F5**b (1–2)^**b**^, **F5**c (1)^**b**^, **F5**d (1–2)^**b**^, **F5**e (1–2)^**b**^
7	19th to 21st	**F1**a (1–8), **F1**b (1–2), **F1**c (1–2), **F1**d (1–2), **F1**e (1–2), **F2**a (1), **F2**b (1), **F2**c (1), **F3**a (1–3), **F3**b (1–3), **F3**c (1–2), **F3**d (1), **F4**a (1–2)^**a**^, **F4**b (1–3)^**a**^, **F4**c (1–2)^**a**^, **F5**a (1–7)^**a**^, **F5**b (1–2)^**a**^, **F5**c (1)^**a**^, **F5**d (1–3)^**a**^, **F5**e (1–2)^**a**^, **F5**f (1–2)^**a**^	**F1**a (1–8), **F1**b (1–2), **F1**c (1–2), **F1**d (1–2), **F1**e (1–2), **F2**a (1), **F2**b (1), **F2**c (1), **F3**a (1–3), **F3**b (1–3), **F3**c (1–2), **F3**d (1), **F4**a (1–2)^**b**^, **F4**b (1–3)^**b**^, **F4**c (1–2)^**b**^, **F5**a (1–7)^**§**^, **F5**b (1–2)^**b**^, **F5**c (1)^**b**^, **F5**d (1–3)^**b**^, **F5**e (1–2)^**b**^, **F5**f (1–2)^**b**^
8	22nd to 24th	**F1**a (1–8), **F1**b (1–2), **F1**c (1–2), **F1**d (1–2), **F1**e (1–2), **F2**a (1), **F2**b (1), **F2**c (1), **F3**a (1–3), **F3**b (1–3), **F3**c (1–2), **F3**d (1), **F4**a (1–2)^**a**^, **F4**b (1–3)^**a**^, **F4**c (1–2)^**a**^, **F4**d (1)^**a**^, **F5**a (1–7)^**a**^, **F5**b (1–2)^**a**^, **F5**c (1)^**a**^, **F5**d (1–3)^**a**^, **F5**e (1–2)^a^, **F5**f (1–4)^a^	**F1**a (1–8), **F1**b (1–2), **F1**c (1–2), **F1**d (1–2), **F1**e (1–2), **F2**a (1), **F2**b (1), **F2**c (1), **F3**a (1–3), **F3**b (1–3), **F3**c (1–2), **F3**d (1), **F4**a (1–2)^**b**^, **F4**b (1–3)^**b**^, **F4**c (1–2)^**b**^, **F4**d (1)^**b**^, **F5**a (1–7)^**b**^, **F5**b (1–2)^**b**^, **F5**c (1)^**b**^, **F5**d (1–3)^**b**^, **F5**e (1–2)^**b**^, **F5**f (1–4)^**b**^

Note: *CG* Control group (dual-task training with variable priority attention), *EG* Experimental group (dual-task training with progression of activities from variable to fixed priority attention); ^a^Secondary cognitive or motor task will always be interspersed with dual tasks with variable priorities; ^b^Secondary cognitive or motor task will always be performed simultaneously with dual tasks with fixed priorities

Participants of both groups must attend at least 75% [26] of the 60-min training sessions, which will occur twice a week for 24 weeks. Each of the 48 group training sessions (maximum of 15 participants in each group) will include: i) a warm-up (10 min) with supervised walking on a flat surface and static postural balance exercises, ii) training (four stations, 10 min each, total of 40 min) for protocol execution, and iii) a cool-down, including breathing exercises and global muscle stretching (10 min). The principles of this training will follow recommendations from previous studies [26–29]. Interventions of each group will be supervised by a physical therapist with experience in ​​dual-task exercises and by four undergraduate students of the final year’s physical therapy course. Adherence monitoring will be done by signature in the presence table in each training session. Concomitant exercise programs for postural balance are not permitted during the trial for both groups.

The dual-task training program is designed based on activities described by Wollesen et al. [29] as well as the studies by Strouwen et al. [30] and Zhao and Pak-Kwongchung [31]. Table 1 presents the conceptual basis that will be used to structure the dual-task protocol training. The progression of the protocol will be based on eight foci (F1–F8), while strategies will progress in intensity and degree of difficulty; the tasks will be determined by associating the respective foci (F1–F8) with the strategies. This will allow for the graduation of the challenge level at each training stage regarding participants’ postural control and gait pattern.

Table 2 shows the methodology that will be applied to the progression of the exercises, structured according to the conceptual basis described in Table 1. This protocol will be performed in a circuit composed of hula hoops, ropes (in a straight line and zigzagging), an agility ladder, traffic cones, steps, cardboard boxes, and other obstacles arranged on the floor (stable surface) or on mattresses (unstable surface), depending on the aim of each training stage. Before starting each session, the instructors will explain all exercises with additional verbal feedback to improve task performance.

### Adverse events

All adverse events will be self-reported by the participant to the principal researcher. An adverse event will be defined as any unfavorable or unintentional health-related event (sign, symptom, syndrome, or disease) that develops or worsens during the study period. These events will be monitored closely until a resolution or stabilization is achieved, or until it has been shown that the study intervention is not the cause of the event. According to the recent epidemiologic data of the Brazilian Longitudinal Study of Aging (ELSI-Brazil) [32], if falls incidences during the training session are greater than 25% with 3% resulting in a hip or femur fracture, the study will be interrupted. The decision to do so will be immediately reported to the research team, and the local research ethics committee will be notified. If there is a fall episode during training that requires medical attention or any other complication during the execution of the study, the mobile emergency response service will be contacted so that the participant can be promptly referred to the local university hospital.

### Outcomes

#### Primary outcome measures

The primary outcome will be the self-selected gait speed under fixed-priority dual tasks [18]. The walking speed was chosen as the primary outcome since it has been reported as an indicator of functional performance in older adults and a good predictor of physical performance [33–35], mortality [35], and falls [36–38].

For gait speed assessment, the participants will be asked to walk a leveled 30-m-long corridor twice (a total of 60 m) at a comfortable speed while barefoot. The initial and final 2 m (positive and negative acceleration, respectively) will not be considered for gait biomechanics analysis. Gait speed assessment will be described in the “Process A” section. This outcome will be assessed at all the time points.

#### Secondary outcome measures

Gait variables, functional mobility and balance tests, reactions time, confidence and fear of falls, quality of life, depression symptoms and fall episodes are the secondary outcomes and will be assessed at all the time points. These secondary outcomes presented in Table 3 were chosen because they represent the functional, biomechanical, and quality of life aspects of patients at risk for falls.

**Table 3:** Schedule of forms and procedures of the study

				−1	0	T1	T2	T3	T4	T5
Activity/ Assessment	CRF (Y/N)	Staff member	Approximate time to complete	Pre-study screening/ consent	Pre-study baseline/ randomization	Study visit 1 immediately post-randomization	Study/ interim visit 2 (3 months)	Study/ interim visit 3 (6 months)	Post-intervention follow-up (3 months)	Post-intervention follow-up (6 months)
Pre-screening consent	N	Study Coordinator	5 min	X						
Screening log	N	Study Coordinator	5 min	X						
Inclusion/ exclusion form	Y	Study Coordinator	N/A	X						
Demographic data	Y	Interviewer	10 min		X					
Anthropometric data	Y	Assessor	5 min		X					
Gait speed, general, temporal and spatial variables	N	Assessor	15 min			X	X	X	X	X
Displacement of the body center of mass during *quasi*-static posture	N	Assessor	15 min			X	X	X	X	X
Functional balance tests	Y	Assessor	15 min			X	X	X	X	X
Mobility test	Y	Assessor	10 min							
Reaction time	Y	Assessor	10 min			X	X	X	X	X
Confidence and fear of falls	Y	Interviewer	10 min			X	X	X	X	X
Quality of life	Y	Interviewer	10 min			X	X	X	X	X
Depression symptoms	Y	Interviewer	10 min			X	X	X	X	X
Fall episodes	Y	Interviewer				Monthly				
Adverse events	Y	Study Coordinator	*N/A*			As needed throughout the protocol				
Adherence to the exercise program	Y	Interviewer				Monthly				

*CRF* Case report form, *N/A* Not applicable

### Participant timeline

The assessments will be performed at T1, T2, T3, T4, and T5. A 14-day window, defined as 7 days before and 7 days after the due date, will be available to complete the assessments.

#### Data collection, management, and analysis

Two assessors (ACS and IFS) will carry out all the data collection, management, and analysis. Both evaluators were submitted to a previous and extensive training protocol for data collection, management, and analysis. Clinical and functional data will be collected by ACS and biomechanics data by IFS. Double data entry will be done interchangeably by both assessors.

#### Process A: biomechanical analysis

Three Physilog® sensors (Gait Up, Lausanne, Switzerland) will be used in this study for biomechanical analysis of all functional activities. Physilog® is an inertial measurement unit (IMU) based on a standalone device (dimensions: 50 mm × 40 mm × 16 mm; weight: 36 g) including a tri-axial accelerometer (MMA7341LT, range ± 3 g, Freescale, Austin, TX, USA), a tri-axial gyroscope (ADXRS, range ± 600°/s, Analog Devices, Norwood, MA, USA), a battery (3.7 V, 595 mAh), a memory unit, and a microcontroller.

To measure gait spatiotemporal and foot clearance variables during the single and dual tasks (under variable and fixed priorities), two IMUs will be attached to the feet’s torso with a neoprene strap. Using a hypoallergenic neoprene belt, a waist-worn sensor will be fixed around the participant’s waist to measure tri-axial acceleration and angular velocity data. Prior to gait data acquisition, the participant will have a period to habituate to the laboratory environment. The participant will be asked to walk in the usual way (as carried out in his/her daily activity) on a 30-m walkway (go and come back) at her/his preferred walking speed and discretion [39, 40]. For the gait analysis, the two first strides performed at the beginning and end of the gait test will be excluded (the positive and negative acceleration phases, respectively) [41].

The tri-axial acceleration and angular velocity data will be acquired using the waist-worn sensor while performing (i) a quasi-static posture during the clinical test of sensory interaction and balance (CTSIB), (ii) the Stroop test during quasi-static posture, (iii) the timed up and go (TUG) test (conventional, manual, and cognitive), (iv) the functional anterior reach test, (v) and the sit-and-up from the floor and from a chair test (five times). Prior to the data acquisition, the evaluator will demonstrate the tests to the participant.

All kinematics data (tri-axial acceleration and angular velocity) will be sampled on an on-board 16-bit analog-digital converter at a sampling frequency of 128 Hz. All signals from the three Physilog® sensors will be synchronized by wireless transmission and recorded on a micro SD card inside the IMU before being transferred to a computer. The waist-worn sensor data will be filtered using a 4-th order infinite impulse response (IIR) low-pass Butterworth filter with a cut-off frequency of 5 Hz [42].

The gait variables acquired by both feet-worn sensors will be analyzed by the gait analyzer software (Gait Up, Lausanne, Switzerland). The complexity of multivariate signals over multiple temporal scales acquired by the waist-worn sensor (during gait and quasi-static conditions) will be analyzed by refined composite multivariate generalized multiscale fuzzy entropy using a Matlab routine (Mathworks, Inc.; Natick, MA) developed by Azami and Escudero [43].

#### Process B: assessment of functional balance, fall events, quality of life, and depression symptoms

The following functional capacity tests will be assessed: gait speed [10], spatiotemporal gait biomechanics, TUG_Conventional_ [44], TUG_Cognitive_ [45], TUG_Manual_ [45], postural balance test [46], sit-and-up (from the floor) [47], sit-and-up (from a chair) [48], anterior functional reach test [49], CTSIB [50], Falls Efficacy Scale–International [51], and Activities-specific Balance Confidence scale [52].

In order to evaluate suggestive depression symptoms and quality of life, the Brazilian Short Form of the Geriatric Depression Scale [53] and the Brazilian Medical Outcomes Study 36-Item Short-Form Health Survey, respectively, will be used [54]. Process B will be carried out by two physical therapists (ACS and IFS) with previous experience in functional balance and emotional and quality of life assessment in older adults.

#### Process C: evaluation of the number of falls

Each patient will receive a diary to record the date, time, place, and reason for a fall as well as any injury or form of treatment after the episode. This diary will be collected monthly until the end of the study.

### Statistical analyses

Statistical analyses will be performed based on the intention-to-treat principle. The independent variables of the clinical trial will be both groups (two levels) and the time, counted in weeks (five levels; T1 to T5).

The pattern of missing data will be previously analyzed [55]. A full description of the reasons for possible sample losses will be presented after the end of the study. Exploratory analyses will be performed to verify the distribution of variables, identification of outliers, missing data, and asymmetries.

Generalized Estimation Equations (EEG) will be used for univariate analyses, considering the factors group (EG and CG) and time (T1, T2, T3, T4 and T5), as well as the interaction effect (time vs. group). The most appropriate GEE model for each variable will be confirmed by considering the measurement scale, the Quasi-likelihood Information Criterion (QIC) values, the working correlation matrix, the data distribution, and the respective log link.

Adjustments for univariate (main effects) and multivariate (interaction effect) comparisons of estimated marginal means (EMM) will be made by the Sidak test.

A 95% confidence interval for the mean difference that lies entirely on the EG side will be considered a superiority result statistically significant.

The Statistical Package for the Social Sciences (SPSS, IBM; v.22.0) will be used for statistical analyses.

### Monitoring

Data monitoring committee (DMC) will be formed by the researchers MMN, and TFAS, which are independent of the sponsor and competing interests. Further details about the DMC can be achieved by sending an e-mail to labiafh.env@upe.br.

MMN and TFAS will compose the interim analyses committee in order to make decisions about study stopping or terminating the trial.

Monthly, data collecting, assessing, reporting, and managing solicited and spontaneously reported adverse events and other unintended effects of trial interventions will be carried out by MMN and TFAS, which are independent of investigators and the sponsor.

## Discussion

### Potential impact and significance of the study

This is the first assessor- and participant-blinded, two-arm, randomized controlled trial with 6 months of intervention and an additional 6-month post-training follow up with the aim of evaluating the effectiveness of training with progression from variable- to fixed-priority instructions on gait biomechanics, postural balance, falls episodes, executive functioning, and quality of life in community-dwelling older adults. Although prior systematic reviews [11, 13, 14] have shown the positive effects of variable- and fixed-priority dual-task training programs on balance performance in older adults, the studies neither considered nor structured a training program that prioritized a progression of dual tasks from variable- to fixed-priority instructions. Taking into account that daily living activities take place concomitantly, such as crossing a street and talking on the cell phone, thinking about a shopping list while walking indoors, etc., it is of paramount importance to develop protocols that combine dual-task training with variable- and fixed-priority instructions to improve the above-mentioned aspect.

If our hypotheses are confirmed, this training protocol can be implemented widely to improve gait speed and postural balance in community-dwelling older adults. While it has a longer training time than most similar studies, the proposed study will allow us to evaluate the behavior of the participants every 3 months. In addition, we will be able to evaluate if the possible effectiveness of this protocol will remain for another 6 months after the end of the training. The results of this study could lead to a reduction in hospital admissions secondary to falls as well as lower direct and indirect costs associated with these fall episodes. This study could contribute to future guidelines on gait and postural balance improvement in older adults.

## Data Availability

Trial results will be made available to participants, healthcare professionals via scientific publication, and activities to specific older adults community groups.

## Abbreviations

CG: Control group
CONSORT: Consolidated standards of reporting trials
CTSIB: Clinical Test of sensory interaction in balance
EG: Experimental group
IIR: Infinite impulse response
IMU: Inertial measurement unit
SD: Solid disk
SPIRIT: Standard protocol items
SPSS: Statistical package for the social sciences
T1: Time 1, baseline
T2: At 3 months of intervention
T3: At the end of the 6 months of intervention
T4: At 3 months post-intervention
T5: At 6 months post-intervention
TUG: Timed up and go

## References

[1] Bloem BR, Valkenburg VV, Slabbekoorn M, Willemsen MD (2001). The multiple tasks test: development and normal strategies. Gait Posture.

[2] Li W, Keegan TH, Sternfeld B, Sidney S, Quesenberry CP, Kelsey JL (2006). Outdoor falls among middle-aged and older adults: a neglected public health problem. Am J Public Health.

[3] Berg WP, Alessio HM, Mills EM, Tong C (1997). Circumstances and consequences of falls in independent community-dwelling older adults. Age Ageing.

[4] Smith E, Cusack T, Blake C (2016). The effect of a dual task on gait speed in community dwelling older adults: A systematic review and meta-analysis. Gait Posture.

[5] Chen HC (1996). AB Schultz, JA Ashton-miller, B Giordani, NB Alexander, and KE Guire. Stepping over obstacles: dividing attention impairs performance of old more than young adults. J Gerontol A Biol Sci Med Sci.

[6] Melzer I, Benjuya N, Kaplanski J (2001). Age-related changes of postural control: effect of cognitive tasks. Gerontology.

[7] Melzer I, Liebermann DG, Krasovsky T, Oddsson LI (2010). Cognitive load affects lower limb force-time relations during voluntary rapid stepping in healthy old and young adults. J Gerontol A Biol Sci Med Sci.

[8] Stelmach GE, Zelaznik HN, Lowe D (1990). The influence of aging and attentional demands on recovery from postural instability. Aging (Milano).

[9] Verghese J, Kuslansky G, Holtzer R, Katz M, Xue X, Buschke H (2007). Walking while talking: effect of task prioritization in the elderly. Arch Phys Med Rehabil.

[10] Silsupadol P, Shumway-Cook A, Lugade V, van Donkelaar P, Chou LS, Mayr U (2009). Effects of single-task versus dual-task training on balance performance in older adults: a double-blind, randomized controlled trial. Arch Phys Med Rehabil.

[11] Ghai S, Ghai I, Effenberg AO (2017). Effects of dual tasks and dual-task training on postural stability: a systematic review and meta-analysis. Clin Interv Aging.

[12] Buragada S, Alyaemmi A, Melam RG, Alghamdi AM (2012). Effect of dual task training (fixed priority-versus-variable priority) for improving balance in older adults. World Appl Sci J.

[13] Howe TE, Rochester L, Jackson A, Banks PM, Blair VA (2007). Exercise for improving balance in older people. Cochrane Database Syst Rev.

[14] Howe TE, Rochester L, Neil F, Skelton DA, Ballinger C (2011). Exercise for improving balance in older people. Cochrane Database Syst Rev.

[15] Boutron I, Altman DG, Moher D, Schulz KF, Ravaud P, CN Group. CONSORT statement for randomized trials of nonpharmacologic treatments: a 2017 update and a CONSORT extension for nonpharmacologic trial abstracts. Ann Intern Med. 2017;(1):167, 40–47. 10.7326/M17-0046 28630973

[16] Chan AW, Tetzlaff JM, Altman DG, Laupacis A, Gotzsche PC, Krleza-Jeric K (2013). SPIRIT 2013 statement: defining standard protocol items for clinical trials. Ann Intern Med.

[17] Chan AW, Tetzlaff JM, Gotzsche PC, Altman DG, Mann H, Berlin JA (2013). SPIRIT 2013 explanation and elaboration: guidance for protocols of clinical trials. BMJ.

[18] Silsupadol P, Lugade V, Shumway-cook A, van Donkelaar P, Chou LS, Mayr U (2009). Training-related changes in dual-task walking performance of elderly persons with balance impairment: a double-blind, randomized controlled trial. Gait Posture.

[19] Perera S, Mody SH, Woodman RC, Studenski SA (2006). Meaningful change and responsiveness in common physical performance measures in older adults. J Am Geriatr Soc.

[20] Faul F, Erdfelder E, Lang A-G, Buchner A (2007). G*power 3: a flexible statistical power analysis program for the social, behavioral, and biomedical sciences. Behav Res Methods.

[21] Azadian E, Torbati HR, Kakhki AR, Farahpour N (2016). The effect of dual task and executive training on pattern of gait in older adults with balance impairment: A Randomized controlled trial. Arch Gerontol Geriatr.

[22] Daly RM, Duckham RL, Tait JL, Rantalainen T, Nowson CA, Taaffe DR, et al. Effectiveness of dual-task functional power training for preventing falls in older people: study protocol for a cluster randomised controlled trial. Trials. 2015;16(120):1-15. 10.1186/s13063-015-0652-y PMC4379606 25872612

[23] Hunt MA, Birmingham TB, Giffin JR, Jenkyn TR (2006). Associations among knee adduction moment, frontal plane ground reaction force, and lever arm during walking in patients with knee osteoarthritis. J Biomech.

[24] Vickers AJ (2006). How to randomize. J Soc Integr Oncol.

[25] Dalum HS, Korsbek L, Mikkelsen JH, Thomsen K, Kistrup K, Olander M (2011). Illness management and recovery (IMR) in Danish community mental health centres. Trials.

[26] Sherrington C (2011). A Tiedemann, N Fairhall, JC close, and SR Lord. Exercise to prevent falls in older adults: an updated meta-analysis and best practice recommendations. N S W Public Health Bull.

[27] Hollman JH, Kovash FM, Kubik JJ, Linbo RA (2007). Age-related differences in spatiotemporal markers of gait stability during dual task walking. Gait Posture.

[28] Wollesen B, Schulz S, Seydell L, Delbaere K. Does dual task training improve walking performance of older adults with concern of falling? BMC Geriatr. 2017; 17;(1):213. 10.1186/s12877-017-0610-5 PMC5594522 28893187

[29] Wollesen B, Voelcker-Rehage C, Willer J, Zech A, Mattes K (2015). Feasibility study of dual-task-managing training to improve gait performance of older adults. Aging Clin Exp Res.

[30] Strouwen C, Molenaar EA, Keus SH, Munks L, Munneke M, Vandenberghe W (2014). Protocol for a randomized comparison of integrated versus consecutive dual task practice in Parkinson's disease: the DUALITY trial. BMC Neurol.

[31] Zhao Y, Pak-Kwong C (2015). A preliminary design for a community-based wxercise program for balance improvement and fall prevention. Int J Sports Phys Educ.

[32] Pimentel WRT, Pagotto V, Stopa SR, Hoffmann MCCL, Andrade FB, Junior PRBS (2018). Falls among Brazilian older adults living in urban areas: ELSI-Brazil. Revista de Saúde Pública.

[33] Langlois JA, Keyl PM, Guralnik JM, Foley DJ, Marottoli RA, Wallace RB (1997). Characteristics of older pedestrians who have difficulty crossing the street. Am J Public Health.

[34] Whitehead C, Miller M, Crotty M (2003). Falls in community-dwelling older persons followinig hip fracture: impact on self-efficacy, balance and handicap. Clin Rehabil.

[35] Hardy CJ, Rejeski WJ (1989). Not what, but how one feels: the measurement of affect during exercise. J Sports Exerc Psychol.

[36] Lopopolo RB, Greco M, Sullivan D, Craik RL, Mangione KK (2006). Effect of therapeutic exercise on gait speed in community-dwelling elderly people: a meta-analysis. Phys Ther.

[37] Daley MJ, Spinks WL (2000). Exercise, mobility and aging. Sports Med.

[38] Rubenstein LZ, Powers CM, MacLean CH (2001). Quality indicators for the management and prevention of falls and mobility problems in vulnerable elders. Ann Intern Med.

[39] Morlock M, Schneider E, Bluhm A, Vollmer M, Bergmann G, Muller V (2001). Duration and frequency of every day activities in total hip patients. J Biomech.

[40] Detrembleur C, De Nayer J, van den Hecke A (2005). Celecoxib improves the efficiency of the locomotor mechanism in patients with knee osteoarthritis. A randomised, placebo, double-blind and cross-over trial. Osteoarthr Cartil.

[41] Malatesta D, Canepa M, Fernandez AM (2017). The effect of treadmill and overground walking on preferred walking speed and gait kinematics in healthy, physically active older adults. Eur J Appl Physiol.

[42] Sucerquia Angela, López José, Vargas-Bonilla Jesús (2017). SisFall: A Fall and Movement Dataset. Sensors.

[43] Azami H, Escudero J (2017). Refined composite multivariate generalized multiscale fuzzy entropy: A tool for complexity analysis of multichannel signals. Physica A.

[44] Podsiadlo D, Richardson S (1991). The timed "up & go": a test of basic functional mobility for frail elderly persons. J Am Geriatr Soc.

[45] Woollacott M, Shumway-Cook A (2002). Attention and the control of posture and gait: a review of an emerging area of research. Gait Posture.

[46] Nascimento MN, Appell IPC, H.J AC (2012). Teste de Equilíbrio Corporal (TEC) para idosos independentes. Revista Portuguesa de Ciências do Desporto.

[47] Brito LB, Ricardo DR, Araujo DS, Ramos PS, Myers J, Araujo CG (2014). Ability to sit and rise from the floor as a predictor of all-cause mortality. Eur J Prev Cardiol.

[48] Jones CJ, Rikli RE, Beam WC (1999). A 30-s chair-stand test as a measure of lower body strength in community-residing older adults. Res Q Exerc Sport.

[49] Duncan PW, Weiner DK, Chandler J, Studenski S (1990). Functional reach: a new clinical measure of balance. J Gerontol.

[50] Cohen H, Blatchly CA, Gombash LL (1993). A study of the clinical test of sensory interaction and balance. Phys Ther.

[51] Camargos FFO, Dias RC, Dias JMD, Freire MTF (2010). Adaptação transcultural e avaliação das propriedades psicométricas da Falls Efficacy Scale – International em idosos brasileiros (FES-I-BRASIL). Revista Brasileira de Fisioterapia.

[52] Marques AP, Mendes YC, Taddei U, Pereira CA, Assumpcao A (2013). Brazilian-Portuguese translation and cross cultural adaptation of the activities-specific balance confidence (ABC) scale. Braz J Phys Ther.

[53] Almeida OP, Almeida SA (1999). Reliability of the Brazilian version of the ++abbreviated form of geriatric depression scale (GDS) short form. Arq Neuropsiquiatr.

[54] Ciconelli RM, Ferraz MB, Santos W, Meinão I, Quaresma MR (1999). Tradução para a língua portuguesa e validação do questionário genérico de avaliação da qualidade de vida SF-36 (Brasil SF-36). Rev Bras Reumatol.

[55] Haukoos JS, Newgard CD (2007). Advanced statistics: missing data in clinical research--part 1: an introduction and conceptual framework. Acad Emerg Med.

